# Visualizing the future: impact of holographic visualization on teaching minimally invasive veneer preparation: a randomized controlled trial

**DOI:** 10.1186/s12909-026-10324-5

**Published:** 2026-09-08

**Authors:** Malin Janson, Maximilian Louis Lederer, Christina Marie Dalhoff, Anna Greta Barbe, Anja Liebermann, Christoph Matthias Schoppmeier

**Affiliations:** 1 Faculty of Medicine, Department of Prosthetic Dentistry, University of Cologne, University Hospital Cologne, Kerpener Str. 32, 50931 Cologne, Germany; 2 Faculty of Medicine, Polyclinic for Operative Dentistry and Periodontology, University of Cologne, University Hospital Cologne, Cologne, Germany

**Keywords:** Holographic visualization, Immersive technologies, Hologram, Spatial ability, Dental education, Prosthodontic education, Veneer preparation, Preclinical training

## Abstract

**Background:**

Minimally invasive veneer preparation in prosthodontic education requires advanced spatial understanding and psychomotor precision. Although immersive technologies are increasingly used in dental education, evidence regarding the educational effectiveness of holographic visualization in prosthodontics remains limited. This randomized controlled trial evaluated the impact of holographic visualization on preparation quality and student-reported educational outcomes during preclinical training in minimally invasive veneer preparation.

**Methods:**

Sixty undergraduate dental students were randomly assigned to receive either conventional two-dimensional photographic visualization (*n* = 30) or holographic three-dimensional visualization (*n* = 30). Following a standardized calibration session and baseline preparation, participants performed a second veneer preparation after exposure to the assigned visualization modality. Preparations were independently assessed by two blinded evaluators using a predefined scoring rubric. Within-group changes were analysed using Wilcoxon signed-rank tests, and between-group differences were assessed using analysis of covariance adjusted for baseline performance. Student-reported outcomes were compared using Mann–Whitney U tests with false discovery rate correction.

**Results:**

Both groups showed significant improvements in preparation quality from baseline to post-intervention (both *p* < 0.001). However, no significant between-group differences were observed (adjusted mean difference: 0.60 points; 95% confidence interval: -0.48 to 1.67; *p* = 0.269). Overall student-reported outcomes were comparable between groups. Spatial understanding showed the largest between-group effect in favour of holographic visualization, although this difference was not significant after correction for multiple comparisons. Holographic visualization was highly accepted by students, and 70% supported its future integration into dental education.

**Conclusions:**

Although holographic visualization did not significantly improve immediate preparation quality compared with conventional two-dimensional photographs, it was highly accepted by students and showed the largest observed effect for perceived spatial understanding. These findings suggest that holographic visualization may primarily support spatial understanding and represent a valuable adjunct for teaching complex three-dimensional concepts in preclinical prosthodontic education.

**Trial Registration:**

German Clinical Trials Register (DRKS), DRKS00040713, prospectively registered on 22 June 2026.

**Supplementary Information:**

The online version contains supplementary material available at https://doi.org/10.1186/s12909-026-10324-5.

## Introduction

The acquisition of precise psychomotor skills represents a major challenge in dental education. Successful performance of many restorative procedures requires not only theoretical knowledge but also the ability to understand complex three-dimensional anatomical relationships and translate them into accurate manual execution.

This challenge is particularly pronounced in minimally invasive veneer preparation. Contemporary restorative concepts emphasize the preservation of healthy tooth structure, and ceramic veneers are considered among the most conservative treatment options for the aesthetic and functional rehabilitation of anterior teeth because of their tooth-preserving properties and favourable long-term survival rates [1–3]. However, preparation depths are frequently limited to only a few tenths of a millimetre, meaning that even minor deviations may result in unnecessary loss of healthy enamel or insufficient material thickness. Consequently, successful execution requires a high degree of spatial understanding and technical precision.

Traditionally, these concepts are taught using textbooks, lectures, photographs, and instructor demonstrations [4]. Nevertheless, these approaches predominantly rely on two-dimensional representations of inherently three-dimensional structures [5]. Students are therefore required to mentally reconstruct spatial relationships, preparation depths, and morphological characteristics from static images. Such cognitive transformations place considerable demands on spatial abilities and may increase cognitive load during learning.

Spatial abilities are widely regarded as an important determinant of anatomical and procedural learning. Previous studies have demonstrated that students with stronger spatial abilities tend to perform better in anatomical learning tasks, while targeted educational interventions may further enhance these abilities [6, 7]. From a theoretical perspective, Cognitive Load Theory proposes that learning can be facilitated when unnecessary extraneous cognitive load is reduced, thereby allowing limited working memory resources to be allocated more effectively to relevant learning processes [8]. Three-dimensional visualization methods may therefore provide a didactic advantage by reducing the mental effort required to transform two-dimensional information into accurate spatial representations.

In recent years, immersive technologies have increasingly been integrated into medical and dental education. A systematic review has demonstrated that virtual reality-based learning environments may improve theoretical knowledge and, in some cases, practical skills compared with conventional teaching approaches [9]. In dentistry, immersive technologies have been investigated in several disciplines. Reymus et al. reported positive student perceptions and improved understanding of root canal anatomy using virtual reality-based instruction [10], while Grad et al. described the educational potential of HoloLens-based augmented reality and three-dimensional reference models [11]. Studies evaluating immersive technologies for restorative preparation techniques have yielded heterogeneous results. Whereas some investigations reported improved preparation performance through virtual reality simulations [12], others observed no significant differences [13] or even inferior outcomes compared with conventional teaching approaches [14]. These inconsistent findings suggest that the educational value of immersive visualization technologies remains incompletely understood.

Among these technologies, holographic visualization represents a potentially promising educational approach [15]. Unlike virtual reality systems requiring head-mounted displays, holographic visualizations project three-dimensional structures into real space and can be viewed simultaneously by multiple learners without additional equipment. This allows collaborative observation and discussion while providing learners with the opportunity to inspect structures from different perspectives. A review of the extant literature reveals that holographic visualizations have been demonstrated to facilitate the clear presentation of three-dimensional information, thereby enhancing education and communication [16]. In the context of minimally invasive veneer preparation, holographic models enable realistic visualization of preparation depths, reduction limits, convergence angles, incisal edge concepts, and morphological details. By directly presenting three-dimensional information, holographic visualization may facilitate spatial understanding and reduce the cognitive effort associated with interpreting conventional two-dimensional images.

Despite these theoretical advantages, evidence regarding the educational effectiveness of holographic visualization in dental education remains scarce. In particular, little is known about whether holographic visualization can improve students’ practical performance and perceived learning outcomes during the acquisition of minimally invasive veneer preparation skills.

Therefore, the aim of this randomized controlled trial was to evaluate the effect of holographic three-dimensional visualization, compared with conventional two-dimensional photographs, on the quality of veneer preparations performed by dental students and on student-reported educational outcomes.

The following null hypotheses were tested:

- There is no difference in improvement of preparation quality between students trained with holographic visualization and those trained with conventional two-dimensional images. FY
- There is no difference in post-intervention preparation quality between groups.
- There is no difference in student-reported educational outcomes between groups.

## Materials and methods

### Study design and ethical approval

This monocentric randomized controlled educational trial was conducted at the Department of Prosthetic Dentistry, University Hospital Cologne, Germany, to investigate the educational effectiveness of different visualization modalities on student learning outcomes and procedural skill acquisition in preclinical dental education. The study was designed and reported in accordance with the Consolidated Standards of Reporting Trials (CONSORT) statement. The study was performed in accordance with the principles of the Declaration of Helsinki and received approval from the Ethics Committee of the Medical Faculty of the University of Cologne (Approval No. 25-1261). Furthermore, the trial was prospectively registered in the German Clinical Trials Register No. DRKS00040713 on 22 June 2026, prior to enrollment of the first participant. Participant recruitment and data collection were conducted from 24 June to 8 July 2026. Written informed consent was obtained from all participants prior to study enrollment.

### Study population

A total of 60 undergraduate dental students enrolled in the preclinical prosthodontic courses (ZWK1 and ZWK2) at the University of Cologne were included in the study. Eligibility criteria comprised an age of at least 18 years, enrollment in one of the designated courses, and provision of written informed consent.

### Calibration session and baseline preparation

At the beginning of the study, all participants attended a standardized 45-minute calibration session. During this session, the theoretical principles of minimally invasive veneer preparation designs (“short wrap”, “medium wrap”, and “long wrap”) were taught using standardized two-dimensional instructional images.

Following the calibration session, all participants performed a veneer preparation of tooth 11 on Frasaco phantom heads equipped with standardized maxillary anterior resin teeth (Frasaco GmbH, Tettnang, Germany). Baseline preparations were conducted before exposure to the study intervention and served as a measure of participants’ initial procedural competence.

### Randomization and intervention

After completion of the baseline preparation, participants were randomly allocated to one of two equally sized study groups (*n* = 30 per group) using the web-based randomization platform Sealed Envelope. Randomization was performed by an independent individual who was not involved in teaching, data collection, or outcome assessment. Group allocation remained concealed from the evaluators throughout the study.

Participants assigned to the control group received standardized two-dimensional photographs of medium-wrap veneer preparations of the adjacent maxillary anterior teeth (12, 21, and 22). Images were captured from multiple clinically relevant perspectives, including frontal, mesial, distal, incisal, and palatal views. Participants assigned to the intervention group received an equivalent holographic three-dimensional visualization of the same preparation designs.

To ensure comparability between study groups, both visualization modalities were based on the identical underlying preparation designs and provided the same morphological information. The educational intervention was designed to facilitate spatial visualization and support the transfer of morphological information to a clinically comparable task. As the target tooth (11) was not included in the instructional material, participants were required to apply the acquired visual information to a new but anatomically analogous preparation task.

**Fig. 1:**
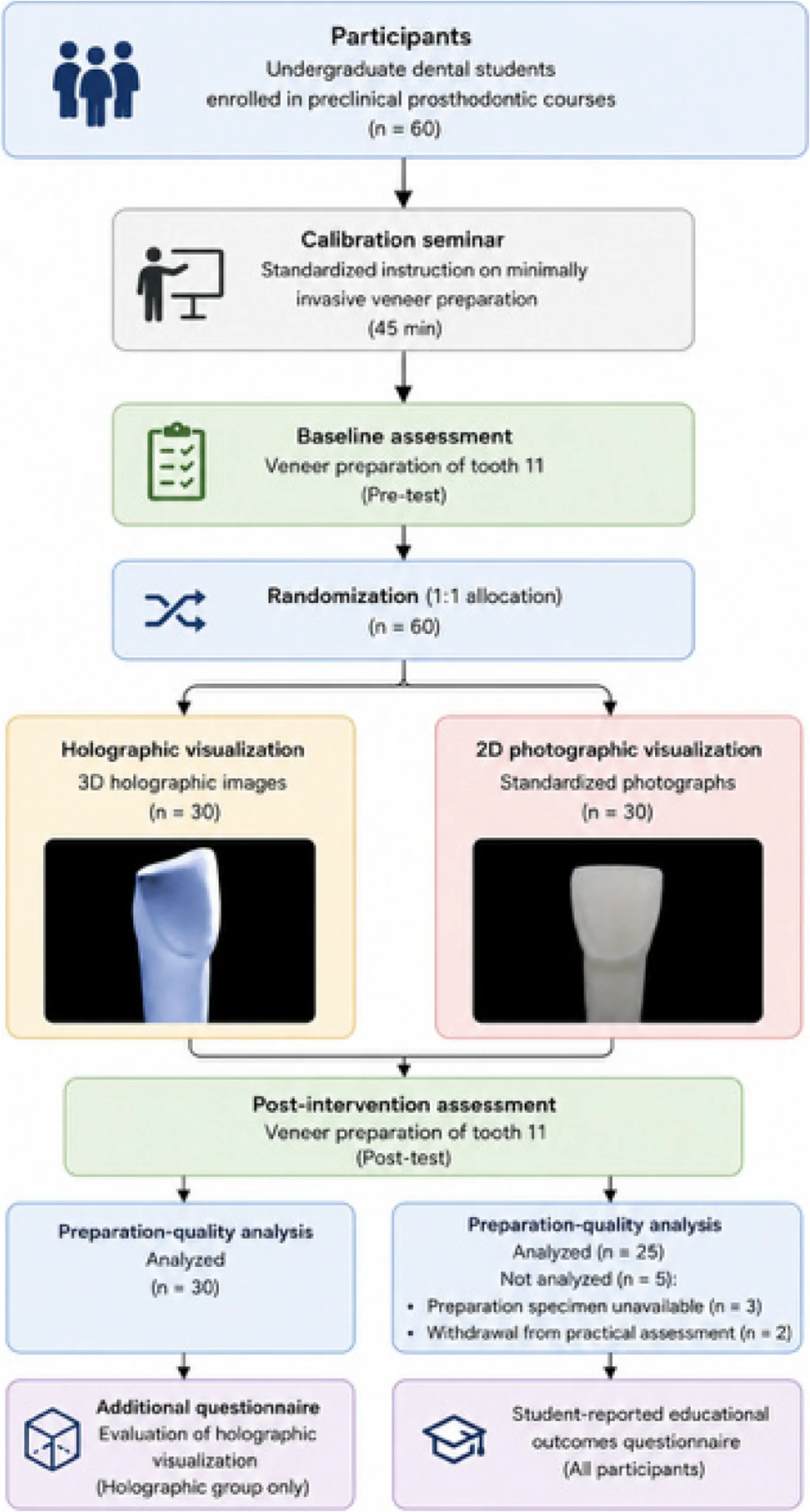
Study flowchart

Participants were allowed to examine the assigned visualization for three minutes. Immediately thereafter, they performed a veneer preparation of tooth 11 on an identical Frasaco phantom model. A maximum preparation time of 60 min was allotted for each participant (Fig. 1).

### Development of the visualization materials

**Fig. 2:**
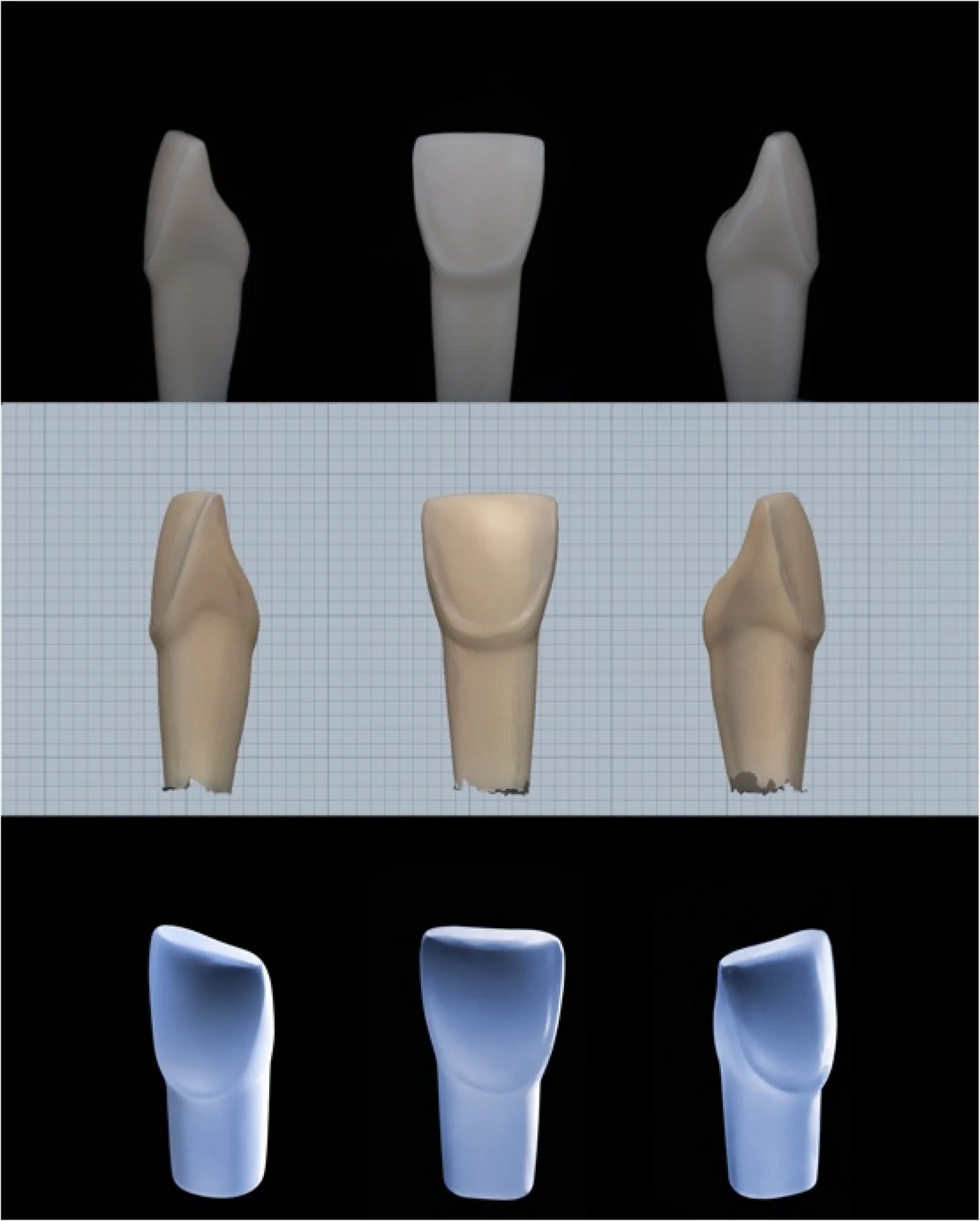
Development of the holographic visualization

Standardized medium-wrap veneer preparations of the maxillary resin anterior teeth 12, 21, and 22 served as the basis for both visualization modalities. For the control condition, high-resolution two-dimensional photographs were obtained from frontal, mesial, distal, incisal, and palatal perspectives. For the holographic visualization, the prepared teeth were digitized using a 3Shape scanner (3Shape A/S, Copenhagen, Denmark). The digital models were further processed in Blender (Blender Foundation, Amsterdam, Netherlands), where material properties were assigned and relevant morphological features, including preparation depth, reduction boundaries, finish-line configuration, and preparation geometry, were visually emphasized. Subsequently, 360° rotational animations were created to enable comprehensive spatial visualization of the preparations (Fig. 2).

The animations were refined using Adobe Premiere Pro (Adobe Inc., San Jose, CA, USA) and exported to the Spin Display application. Final visualization was achieved using a volumetric 3D projector (Dongguan Fankai Hardware Products Co., Guangdong, China) with a projection diameter of 52 cm, mounted on a 120-cm-high tripod, allowing three-dimensional presentation of the prepared teeth in free space without the need for a headset or special glasses. The visualization consisted of a predefined 360° rotational animation. Participants observed the animation passively and could not interact with or manually control the three-dimensional model.

### Assessment of preparation quality

Baseline and post-intervention preparations were independently evaluated by two blinded dentists experienced in the assessment of tooth preparations and indirect restorations. The assessment rubric was developed specifically for this study based on established principles of minimally invasive veneer preparation comprising seven criteria: substance removal according to specifications, chamfer width, incisal reduction, maintenance of the tooth axis, rounding of preparation edges, smoothness of the preparation surface, and finish-line configuration. Each criterion was scored on a three-point scale (0–2 points), with higher scores indicating greater adherence to the predefined preparation criteria, resulting in a maximum total score of 14 points. Objective preparation quality was used as a measure of procedural learning performance and practical skill acquisition. To determine the educational impact of the visualization modality, both within-group improvements between baseline and post-intervention preparations and between-group differences in performance change were analysed. Inter-rater reliability between evaluators was assessed using the intraclass correlation coefficient (ICC).

### Subjective evaluation

Following completion of the practical exercise, participants completed an anonymous questionnaire. The questionnaire was developed specifically for this study and was informed by educational quality principles advocated by the Association for Dental Education in Europe (ADEE). The questionnaire items were reviewed by experienced dental educators for content relevance, clarity, and comprehensibility prior to study implementation. No formal pilot testing was performed. The questionnaire was designed to assess student-related outcomes (SROs) associated with the assigned visualization modality, including understanding of preparation objectives, comprehensibility of the educational content, spatial understanding, procedural confidence, preparedness for independent task performance, and transfer of learning. Responses were recorded using 11-point visual analogue scales ranging from 0 (“strongly disagree”) to 10 (“strongly agree”). The questionnaire was administered in German. For reporting purposes, the questionnaire items were translated into English and are presented in Table 1 together with the corresponding response scales. Participants assigned to the holographic visualization group additionally completed a supplementary questionnaire evaluating perceived innovativeness, usability, technical implementation, potential educational applications, adverse effects, and future integration of holographic technology into dental education. This supplementary questionnaire was likewise administered in German and was translated into English for reporting purposes (Supplementary Table S1).

**Table 1:** Educational evaluation questionnaire

1. The visualization helped me better understand the objectives of the veneer preparation.
2. The educational content was communicated more clearly through the visualization.
3. I was able to accurately perceive the spatial dimensions and extent of tooth reduction.
4. I was able to accurately perceive the spatial dimensions and extent of tooth reduction.
5. Following the learning session, I felt better prepared to perform a veneer preparation independently.
6. The visualization helped me transfer the observed preparation principles to a comparable clinical task.

### Statistical analysis

An a priori sample-size calculation was performed based on the primary outcome of preparation quality. Assuming a medium effect size (Cohen’s d = 0.6), a two-sided significance level of α = 0.05, and a statistical power of 80%, a minimum sample size of 45 participants (approximately 22–23 per group) was required. The target sample size was set at 64 participants to account for potential dropouts. Statistical analyses were performed using R Studio. Statistical significance was set at α = 0.05.

For objective preparation quality, inter-rater reliability was assessed using the intraclass correlation coefficient (ICC; two-way random-effects model, absolute agreement). The mean score of both blinded raters was used for all subsequent analyses. Within-group changes from baseline to post-intervention were evaluated using the Wilcoxon signed-rank test. The primary between-group analysis was performed using analysis of covariance (ANCOVA), modelling post-intervention scores as a function of group while adjusting for baseline performance. Robustness was assessed using a linear mixed-effects model and sensitivity analyses based on Mann-Whitney U and Welch’s t-tests of change scores. Criterion-level changes were compared using Mann-Whitney U tests with Benjamini-Hochberg false discovery rate (FDR) correction for multiple comparisons.

Student-reported outcomes were analyzed as ordinal data. Between-group differences were assessed using two-sided Mann-Whitney U tests, with Benjamini-Hochberg FDR correction applied across the six questionnaire items. The holographic add-on questionnaire was analyzed descriptively. Effect sizes were reported as r (Z/√N) and Cliff’s delta.

## Results

### Participant flow and inter-rater reliability

Of the 60 randomized participants (30 per group), complete preparation-quality data were available for 25 participants in the control group and all 30 participants in the holographic group. Preparation-quality data were unavailable for five participants in the control group due to withdrawal from the practical assessment or unavailable preparation specimens. These reasons were considered unrelated to group allocation or preparation performance. Agreement between the two blinded evaluators was excellent (ICC[A, k] = 0.976; 95% CI: 0.970–0.980).

### Objective preparation quality

**Fig. 3:**
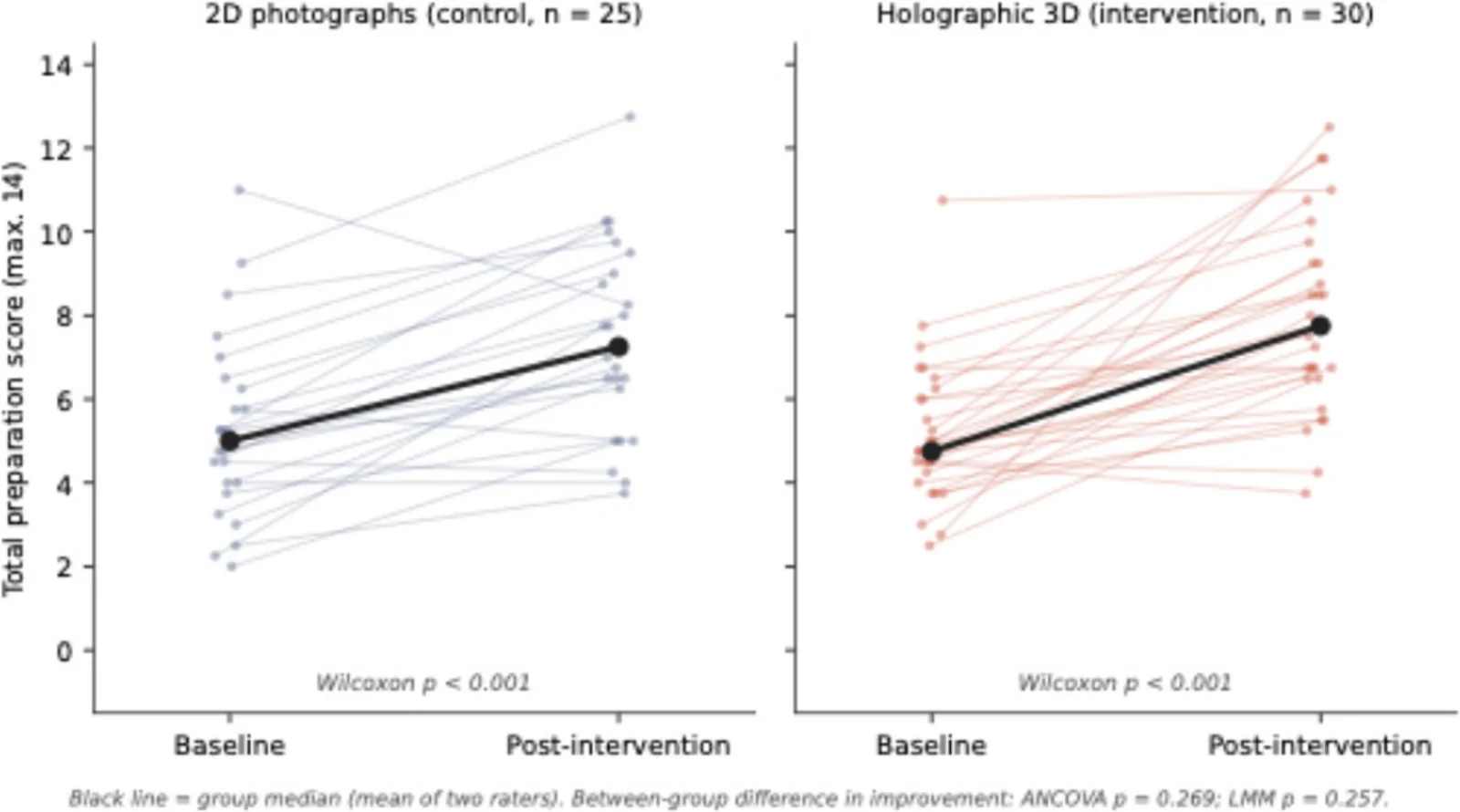
Changes in total preparation scores from baseline to post-intervention

Both groups showed significant improvements in veneer preparation quality from baseline to post-intervention assessment (Table 2; Fig. 3). In the control group, median total preparation scores increased from 5.00 (IQR 4.0-6.2) to 7.25 (IQR 6.2-9.0; *p* < 0.001; *r* = 0.76), whereas scores in the holographic group increased from 4.75 (IQR 4.3-6.0) to 7.75 (IQR 6.6–9.2; *p* < 0.001; *r* = 0.84). The corresponding mean improvements were 2.17 (SD 1.83) and 2.82 (SD 2.31) points, respectively.

**Table 2:** Total preparation scores at baseline and post-intervention

Group	*n*	Baseline Md (IQR)	Baseline M (SD)	Post Md (IQR)	Post M (SD)	Δ Md (IQR)	Δ M (SD)	Wilcoxon *p*	*r* (within)
2D photograph	25	5.00 (4.0–6.2)	5.26 (2.18)	7.25 (6.2–9.0)	7.43 (2.27)	2.50 (1.2–3.2)	2.17 (1.83)	< 0.001	0,76
Holographic 3D	30	4.75 (4.3–6.0)	5.14 (1.66)	7.75 (6.6–9.2)	7.96 (2.25)	2.38 (1.3–4.2)	2.82 (2.31)	< 0.001	0,84

The between-group difference in improvement was not statistically significant. In the baseline-adjusted ANCOVA, the holographic group achieved post-intervention scores that were on average 0.60 points higher than the control group (95% CI: -0.48 to 1.67; *p* = 0.269). Comparable findings were obtained in the linear mixed-effects model (time × group interaction: b = 0.65; 95% CI: -0.47 to 1.77; *p* = 0.257) and in sensitivity analyses using Mann-Whitney U testing (U = 337.5; *p* = 0.531; Cliff’s δ = 0.10) and Welch’s t-test (t = 1.16; *p* = 0.252) (Table 2).

**Fig. 4:**
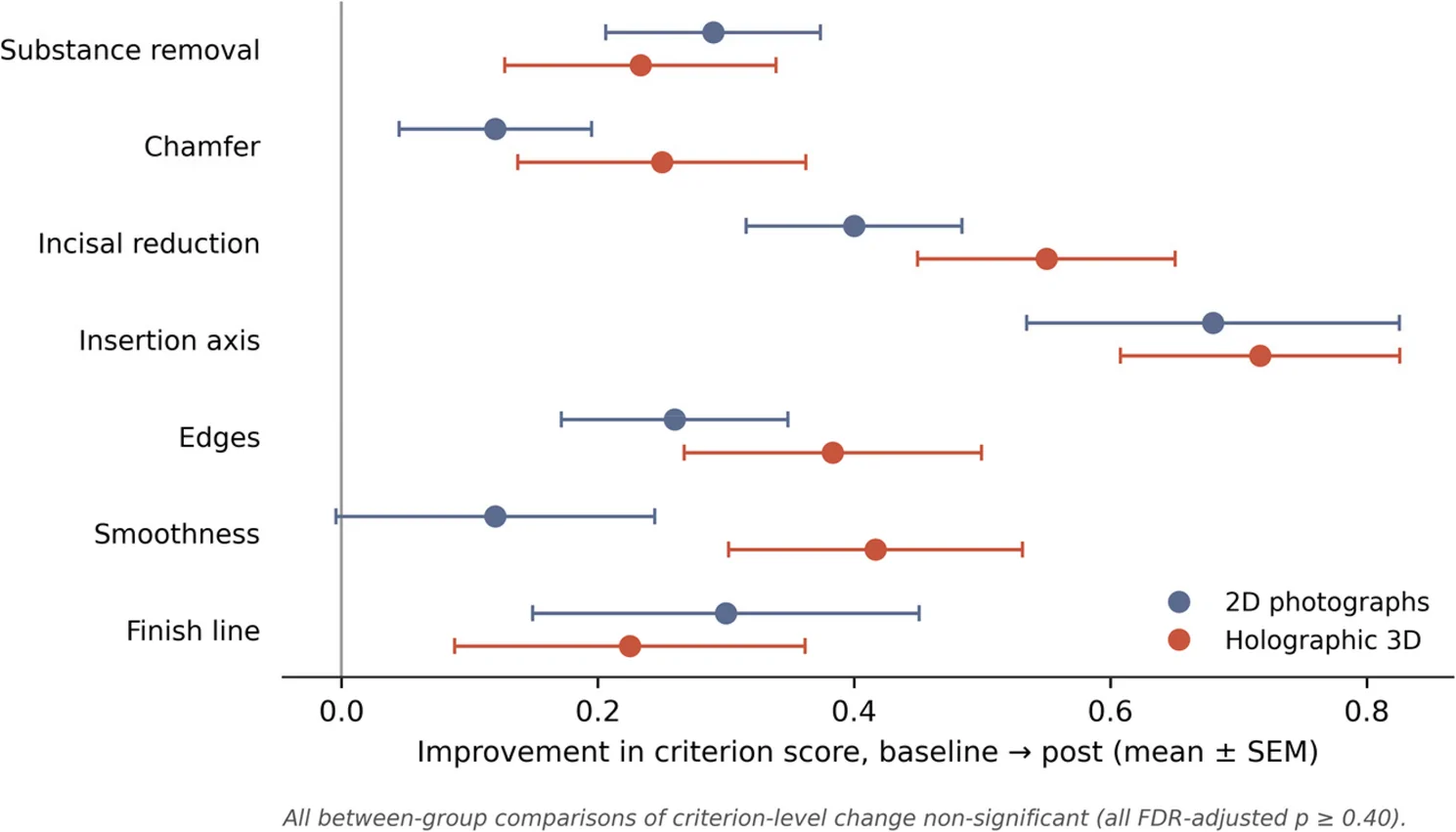
Criterion-level improvement in preparation quality

At the criterion level, improvements were comparable between groups for all seven assessment criteria (all FDR-adjusted *p* ≥ 0.40; Fig. 4). Among the objective preparation-quality criteria, the largest numerical between-group difference was observed for smoothness.

### Student-reported educational outcomes

Questionnaire analyses included 26 participants in the control group and 30 participants in the holographic group. One participant who was excluded from the preparation-quality analysis nevertheless completed the questionnaire and was therefore included in the student-reported outcome analyses.

Overall evaluation scores were comparable between groups (median 40 [IQR 37–46] vs. 41 [IQR 34–48]; *p* = 0.850; *r* = 0.03) (Tables 3 and 4).

**Table 3:** Student-reported educational outcomes by study group

Item	2D photographs (*n* = 26)			Holographic 3D (*n* = 30)		
	Median (IQR)	Mean	SD	Median (IQR)	Mean	SD
E01 Understanding of objectives	7 (7–8)	7,19	1,92	8 (6–8)	7,03	2,06
E02 Comprehensibility	7 (6–8)	7,23	1,42	7 (6–8)	6,81	2,16
E03 Spatial understanding	6 (4–7)	5,62	2,19	7 (6–8)	6,83	1,81
E04 Procedural confidence	7 (5–8)	6,38	1,96	6 (5–8)	6,23	2,13
E05 Preparedness	7 (6–8)	6,77	2,21	7 (5–8)	6,71	2,09
E06 Transfer of learning	7 (6–8)	6,96	2,03	8 (6–9)	7,11	2,34
Total score (0–60)	40 (37–46)	40,15	8,55	41 (34–48)	40,72	9,61

**Table 4:** Between-group comparison of student-reported educational outcomes

Item	Mann-Whitney U	*p* (uncorrected)	*p* (FDR-adjusted)	Effect size *r*	Cliffs δ	Shapiro–Wilk *p* (2D	Shapiro–Wilk *p* (Holo)	Significant (FDR)
E01 Understanding of objectives	396,5	0,921	0,921	0,01	-0,02	0,037	0,01	No
E02 Comprehensibility	419	0,634	0,917	0,06	-0,07	0,058	0,125	No
E03 Spatial understanding	264	0,036	0,215	0,28	0,32	0,056	0,054	No
E04 Procedural confidence	408,5	0,764	0,917	0,04	-0,05	0,175	0,103	No
E05 Preparedness	413	0,708	0,917	0,05	-0,06	0,013	0,123	No
E06 Transfer of learning	357	0,588	0,917	0,07	0,08	0,003	0,006	No
Total score (0–60)	378	0,852	-	0,03	0,03	0,131	0,312	No

Among the student-reported outcomes, spatial understanding showed the largest between-group effect (Cliff’s δ = 0.32), favoring holographic visualization (Tables 3 and 4; Fig. 4). Participants in the holographic group reported higher spatial comprehension of the preparation design (median 7 [IQR 6–8]) than participants receiving conventional photographs (median 6 [IQR 4–7]; uncorrected *p* = 0.036; *r* = 0.28). However, this difference was no longer statistically significant after Benjamini–Hochberg correction for multiple testing (adjusted *p* = 0.215).

**Fig. 5:**
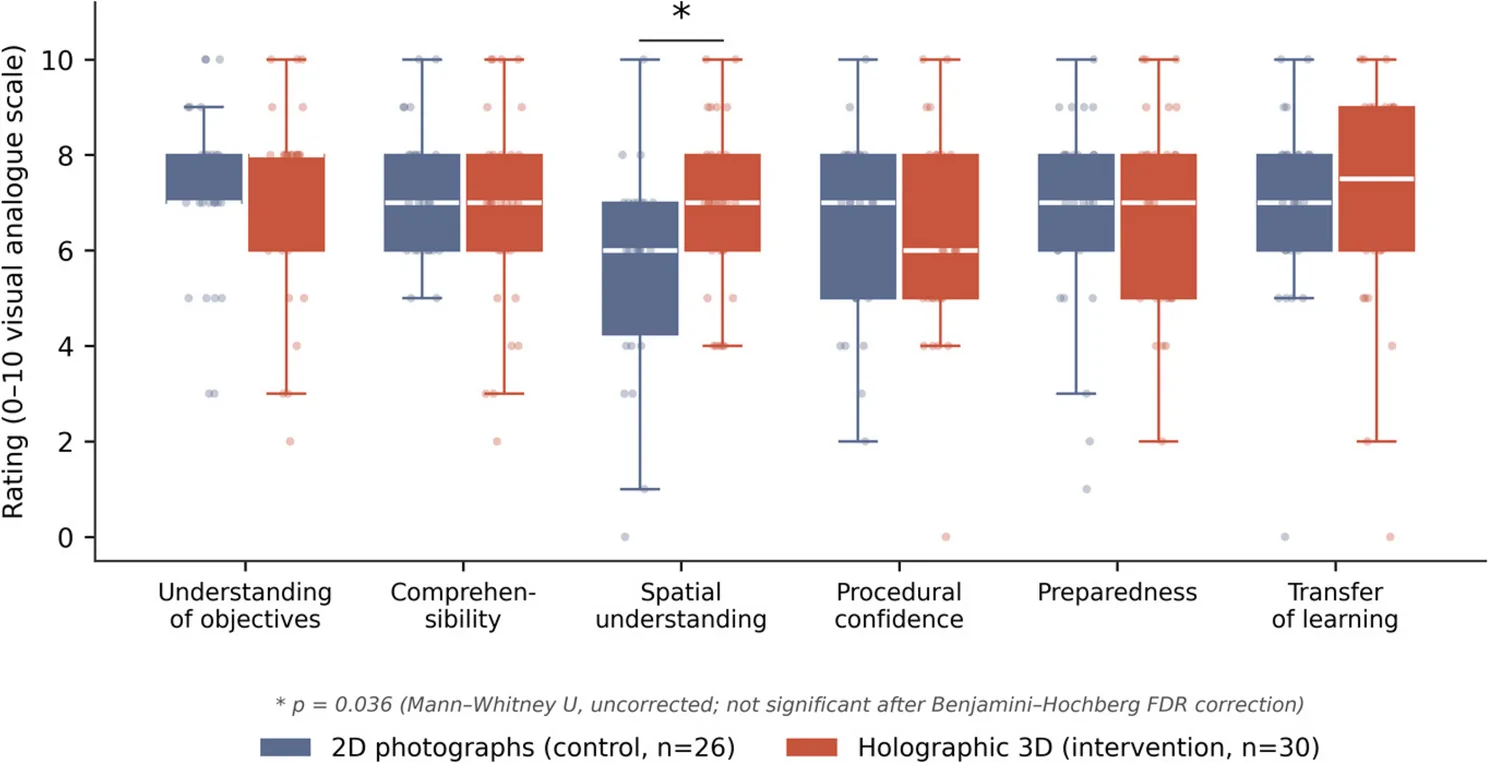
Student-reported educational outcomes

No significant differences were observed for understanding of objectives (E01), comprehensibility (E02), procedural confidence (E04), preparedness (E05), or transfer of learning (E06), with all adjusted p-values ≥ 0.917 and negligible effect sizes (|Cliff’s δ| ≤ 0.08) (Table 4; Fig. 5, Supplementary Figure S1).

### Evaluation of holographic visualization

Within the holographic group, perceived innovativeness was rated highly for both prosthodontic education (median 7, IQR 6–8) and other areas of dental education (median 7, IQR 5–8). Educational potential for patient communication was rated particularly positively (median 8, IQR 5–9), while usability was rated favorably (median 7, IQR 6–8) (Supplementary Table 2).

Twenty-one of 30 respondents (70%) indicated that they would welcome future integration of holographic visualization into dental education, whereas four participants (13%) reported adverse effects during use (Supplementary Table S2).

## Discussion

Both visualization modalities resulted in significant improvements in veneer preparation quality, indicating that the instructional content effectively enhanced students’ performance irrespective of the visualization method employed. Although participants exposed to holographic visualization demonstrated numerically greater improvements in preparation scores, no statistically significant differences in objective preparation quality were observed between the holographic and conventional two-dimensional visualization groups.

Accordingly, none of the three predefined null hypotheses were rejected: no significant between-group differences were observed in the improvement in preparation quality, post-intervention preparation quality, or student-reported educational outcomes after correction for multiple comparisons.

Overall, the findings suggest that holographic visualization may primarily support cognitive aspects of learning, particularly spatial understanding, whereas improvements in psychomotor performance may require repeated practice and longer periods of training. Holographic visualization was associated with the largest observed effect for spatial understanding, suggesting a potential advantage in the perception of three-dimensional preparation geometry. Although this effect did not remain statistically significant after correction for multiple testing, it aligns closely with the theoretical rationale underlying the intervention. In addition, the technology was highly accepted by participants, with most students supporting its future integration into dental education. Taken together, these findings indicate that holographic visualization represents a feasible and well-received educational tool for supporting the acquisition of complex three-dimensional concepts in preclinical prosthodontic education.

### Objective learning outcomes and spatial understanding

The overall pattern of results suggests that the educational effects of holographic visualization are more closely related to cognitive learning processes than to immediate psychomotor execution. Although objective preparation quality did not differ significantly between groups, spatial understanding showed the largest between-group effect, indicating that holographic visualization may facilitate the formation of three-dimensional mental representations. Previous studies on immersive educational technologies have reported similarly heterogeneous findings. While enhanced visualization alone has not consistently translated into superior practical performance, more interactive approaches have demonstrated measurable educational benefits [5, 17]. For example, Zingg et al. reported improved theoretical and practical anatomy performance using an interactive HoloLens-based application, highlighting the importance of instructional design in addition to the visualization modality itself [18]. Several factors may explain why no significant between-group difference in preparation quality was observed. First, all participants received a standardized 45-minute calibration session before the intervention, which may already have produced substantial learning gains. Second, exposure to the visualization material was limited to approximately three minutes, which is unlikely to be sufficient for cognitive advantages to translate into measurable psychomotor improvements. Finally, preparation quality reflects both conceptual understanding and manual execution, whereas the holographic intervention was primarily designed to enhance spatial perception.

This interpretation is consistent with Cognitive Load Theory and the Cognitive Theory of Multimedia Learning [8, 19]. By presenting preparation geometry directly in three dimensions, holographic visualization may reduce the mental effort required to reconstruct spatial information from conventional two-dimensional images. Such cognitive benefits, however, are not expected to translate immediately into superior manual performance, as psychomotor skill acquisition depends on repeated practice, feedback, and motor consolidation. The discrepancy between improved perceived spatial understanding and unchanged preparation quality therefore suggests that holographic visualization primarily influences cognitive aspects of learning that are only indirectly captured by procedural outcome measures.

The questionnaire findings support this interpretation. Among all student-reported outcomes, spatial understanding showed the largest between-group effect, whereas no comparable differences were observed for procedural confidence or preparedness. Although this trend did not remain statistically significant after correction for multiple testing, it is consistent with the proposed cognitive mechanism underlying holographic visualization. Minimally invasive veneer preparation is inherently spatial and requires students to understand reduction depth, preparation margins, surface transitions, and tooth morphology. Unlike conventional photographs, holographic visualizations allow learners to inspect preparation geometry from multiple perspectives while preserving depth cues. Previous studies have suggested that such visualizations facilitate the construction of accurate mental representations of complex anatomical structures and preparation designs [20, 21].

### Student acceptance and educational value

A notable finding of the present study was the high level of student acceptance of holographic visualization. Participants rated the technology favorably with regard to innovativeness, usability, and educational value, and 70% indicated that they would support its future integration into dental education. These findings are consistent with previous investigations of virtual, augmented, and mixed reality technologies in dental education, which have repeatedly reported high levels of learner satisfaction, motivation, and perceived usefulness [5, 12].

Similar observations have been reported in studies investigating holographic and mixed-reality technologies in medical education [22]. High levels of acceptance, learner engagement, and willingness to incorporate such technologies into future educational settings have been consistently described, particularly in anatomy education and simulation-based training [18, 23]. Recent reviews further suggest that learners generally perceive holographic visualization as an innovative and valuable complement to conventional teaching approaches, even when objective learning benefits are not consistently demonstrated [23]. These findings further support the notion that immersive three-dimensional learning environments are highly appreciated by learners and may enrich the overall learning experience [18].

Importantly, educational technologies should not be evaluated solely on the basis of immediate performance outcomes [19]. In addition to knowledge acquisition, they may contribute to learner engagement, curiosity, confidence, and satisfaction, all of which are important determinants of successful learning [4]. The consistently positive perception of holographic visualization observed in the present study suggests that such technologies may enhance the overall learning experience even when measurable improvements in examination or performance outcomes are not immediately apparent.

The strong willingness of participants to use holographic visualization in the future further indicates that students perceive this technology as a meaningful supplement rather than a replacement for established teaching methods. Consequently, its educational value may lie less in substituting conventional instructional approaches and more in complementing existing learning resources by providing an additional three-dimensional perspective on complex clinical procedures.

### Limitations

Several limitations should be considered when interpreting the present findings. First, the sample size available for the primary outcome was relatively small, with complete preparation-quality data available for 55 participants. Although a numerical trend in favor of holographic visualization was observed, the study may have been underpowered to detect small-to-moderate between-group differences.

Second, exposure to the visualization material was limited to approximately three minutes. While this duration was sufficient to familiarize participants with the preparation design, it may have been insufficient for potential advantages of holographic visualization to translate into measurable differences in psychomotor performance. The three-minute exposure was likely adequate to support initial spatial orientation but probably too brief for improvements in cognitive processing to manifest as measurable manual performance. Since psychomotor skill acquisition typically depends on repeated practice, feedback, and consolidation, immediate preparation quality may not represent the most sensitive endpoint for detecting early educational benefits of holographic visualization.

More prolonged or repeated exposure may be necessary to fully exploit the educational potential of immersive visualization technologies. Third, the study was conducted at a single academic institution and investigated only one preparation task, namely a minimally invasive medium-wrap veneer preparation. The generalizability of the findings to other educational settings, student populations, or clinical procedures therefore remains uncertain. Finally, only short-term outcomes were assessed. Neither knowledge retention nor long-term skill acquisition was evaluated. It therefore remains unclear whether the observed differences in perceived spatial understanding may translate into advantages that become apparent only after repeated practice or longer periods of learning.

### Future directions

Future research should investigate larger and more diverse student cohorts, ideally in multicenter settings, to confirm the trends observed in the present study. In addition, longer and repeated exposure to holographic visualizations may provide a more realistic representation of how such technologies would be implemented within dental curricula and may reveal educational benefits that were not detectable within the present study design.

Further investigations should also examine more complex clinical procedures that place greater demands on spatial reasoning and decision-making. Particular emphasis should be placed on outcome measures assessing spatial cognition, morphological understanding, and long-term retention, as these may be more sensitive to the educational mechanisms through which holographic visualization is expected to operate. Finally, the integration of holographic visualization into broader digital learning environments, including augmented and mixed reality platforms, represents a promising area for future educational research.

## Conclusions

Within the limitations of this randomized controlled trial, holographic visualization was highly accepted by students and showed indications of improved perceived spatial understanding. Although no significant advantage in immediate preparation quality compared with conventional two-dimensional photographic visualization was observed, the findings suggest that its primary educational value may lie in facilitating spatial understanding rather than immediate psychomotor performance. As a feasible and well-accepted adjunct to conventional dental education, holographic visualization may be particularly valuable for supporting the acquisition of complex three-dimensional concepts in preclinical prosthodontic training. Future studies should investigate whether repeated exposure allows these potential cognitive advantages to translate into improved procedural performance.

## Supplementary Information

Supplementary Material 1.

Supplementary Material 2.

## Data Availability

The datasets of the current study are available from the corresponding author on reasonable request.

## Abbreviations

FDR: False discovery rate
SEM: Standard error of the mean
