# Supplementary material for: Visualizing the future: impact of holographic visualization on teaching minimally invasive veneer preparation: a randomized controlled trial
Source: BMC Med Educ. 2026 Sep 8;26:1447. doi: 10.1186/s12909-026-10324-5 (PMC13556104; doi:10.1186/s12909-026-10324-5)
Supplement: Supplementary file 1 [file 12909_2026_10324_MOESM1_ESM.docx]

1. How innovative do you consider holographic technology for prosthodontic education?

2. How innovative do you consider holographic technology for other dental disciplines (e.g., orthodontics, restorative dentistry, oral surgery)?

3. In which field of dentistry do you perceive the greatest potential for holographic technology?

4. How innovative do you consider holographic technology for patient education and communication?

5. I was able to use and understand the holographic visualization without difficulty.

6. How would you rate the size of the holographic representation?

7. How would you rate the duration of the holographic animation?

8. How would you rate the technical implementation of the holographic visualization?

9. Do you have suggestions for improving the technical implementation of the holographic visualization?

10. Did you experience any physical discomfort (e.g., headache, dizziness, eye strain) during or after viewing the hologram?

11. Would you recommend the continued use of holographic visualizations in dental education? If yes, from which semester onwards?

**Supplementary Table S1. Holographic Visualization Questionnaire administered to participants in the intervention group.**
