# Supplementary material for: Visualizing the future: impact of holographic visualization on teaching minimally invasive veneer preparation: a randomized controlled trial
Source: BMC Med Educ. 2026 Sep 8;26:1447. doi: 10.1186/s12909-026-10324-5 (PMC13556104; doi:10.1186/s12909-026-10324-5)
Supplement: Supplementary file 2 [file 12909_2026_10324_MOESM2_ESM.docx]

| Item | n | Median (IQR) | Mean | SD |
| --- | --- | --- | --- | --- |
| Innovation (prosthodontics) | 29 | 7 (6-8) | 6,83 | 1,75 |
| Innovation (other subjects) | 29 | 7 (5-8) | 6,66 | 1,99 |
| Patient education | 29 | 8 (5-9) | 7,33 | 2,35 |
| Usability / coping | 29 | 7 (6-8) | 7,07 | 2,05 |
| Technical implementation | 28 | 6 (5-7) | 6,04 | 2,35 |
| Device size (adequacy) | 28 | 3 (3-3) | 2,96 | 0,74 |
| Demonstration duration | 28 | 3 (3-4) | 3,21 | 0,57 |

**Supplementary Table S2.** Participant evaluation of the holographic visualization system.
